# Supplementary figures and images for: Development and characterization of a scalable calcium imaging assay using human iPSC-derived neurons
Source: Front Cell Neurosci. 2025 Dec 15;19:1701907. doi: 10.3389/fncel.2025.1701907 (PMC12745381; doi:10.3389/fncel.2025.1701907)

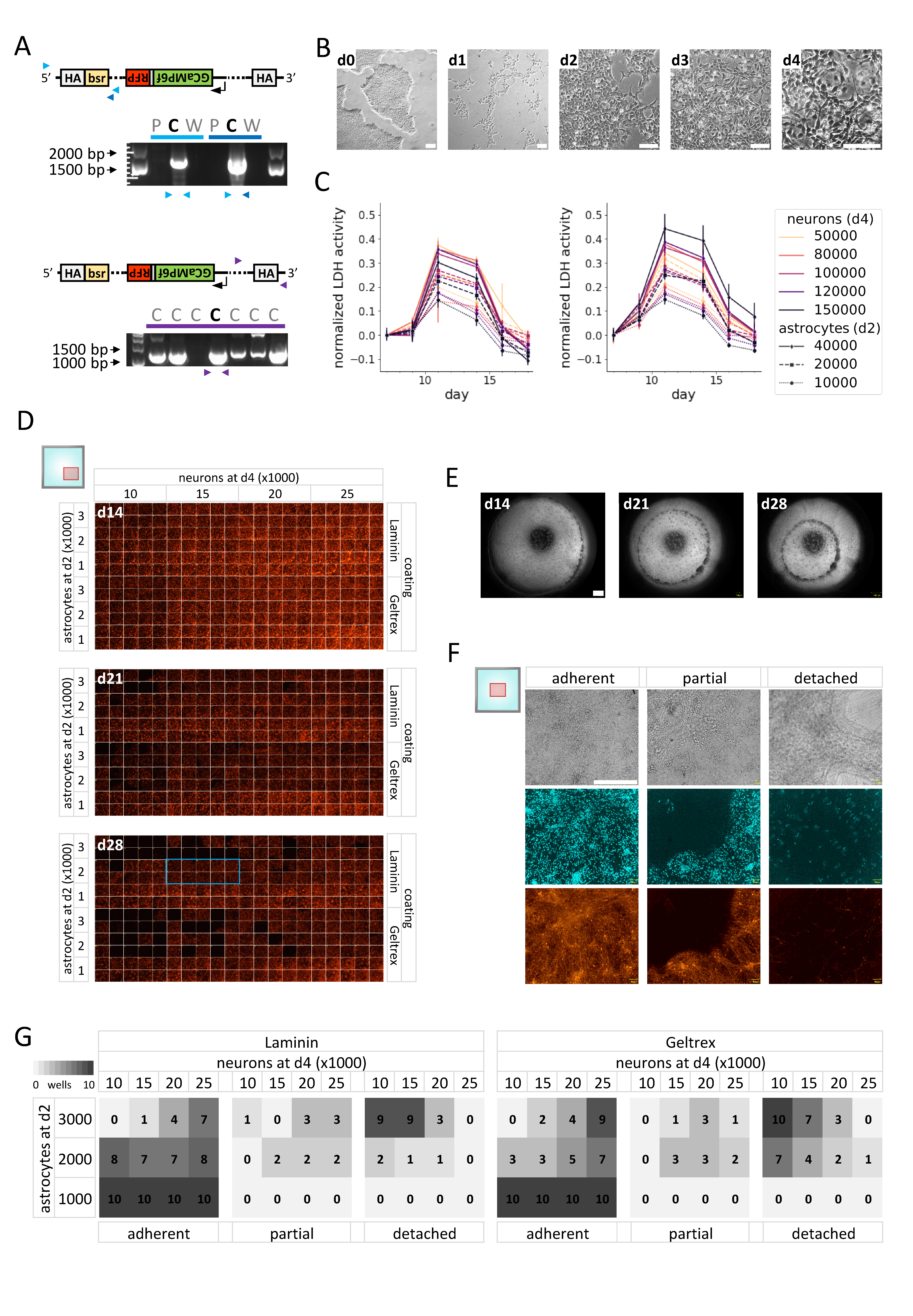

Supplement: Supplementary file 4 [file Image_1.TIF]

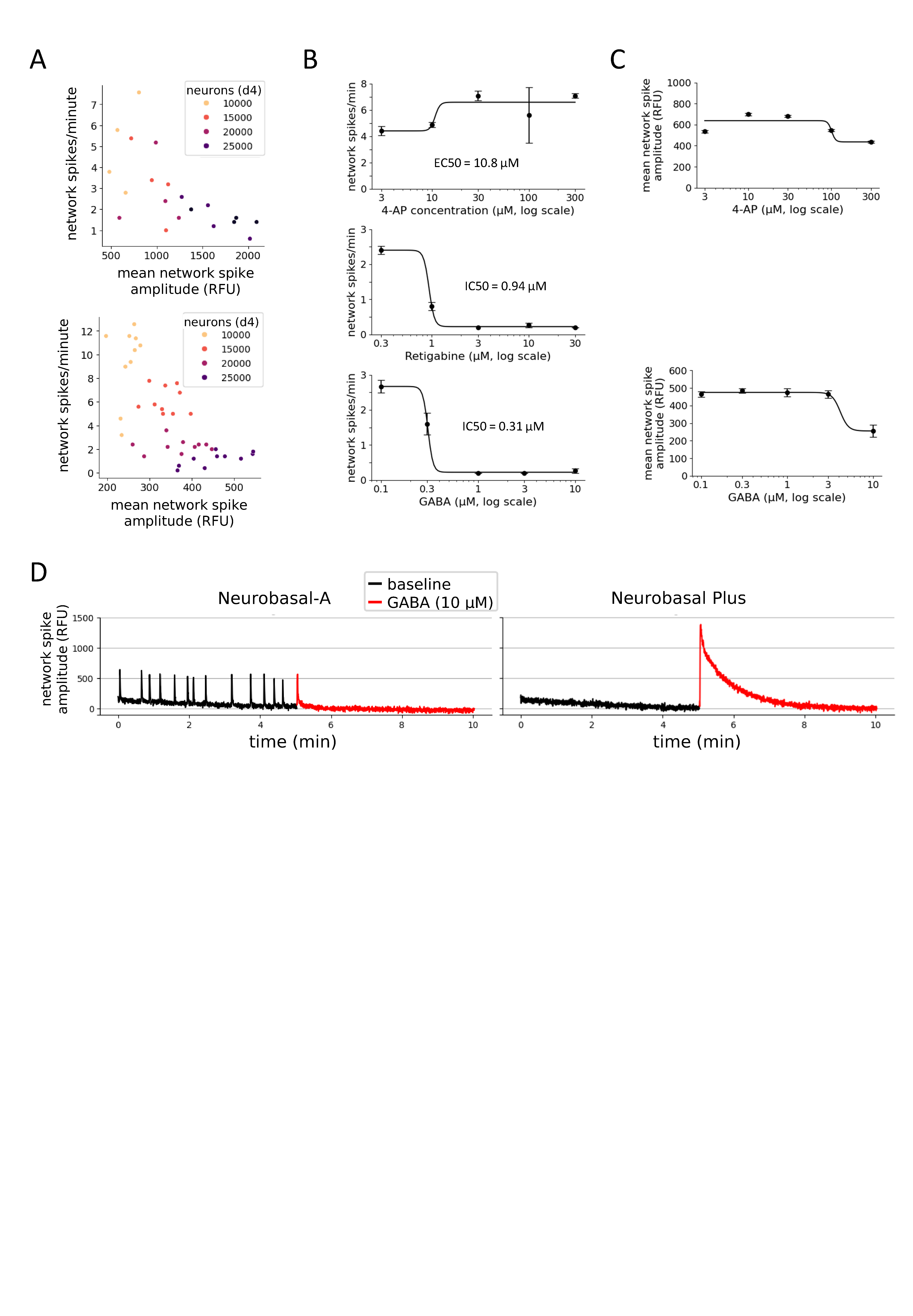

Supplement: Supplementary file 5 [file Image_2.TIF]

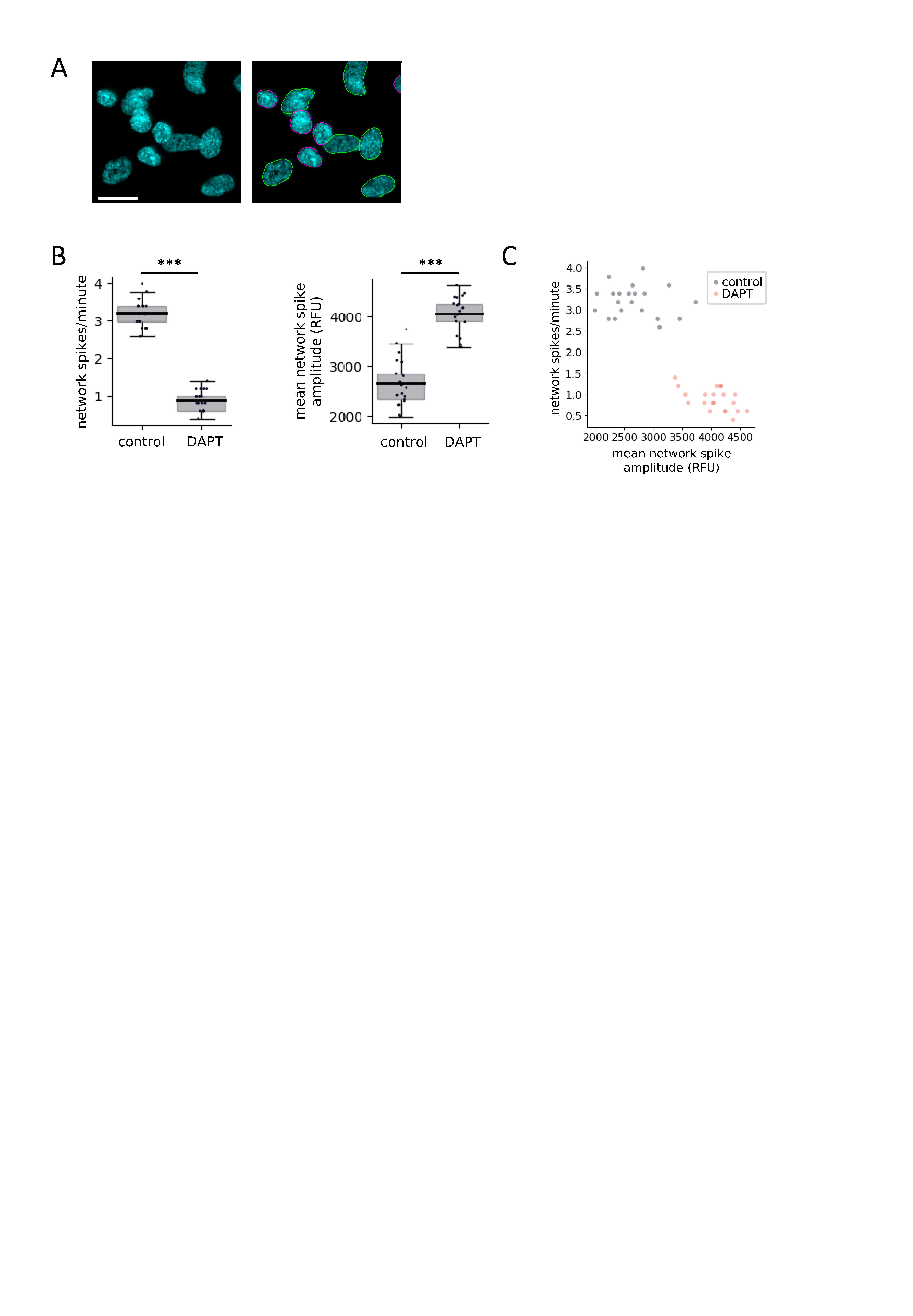

Supplement: Supplementary file 6 [file Image_3.TIF]

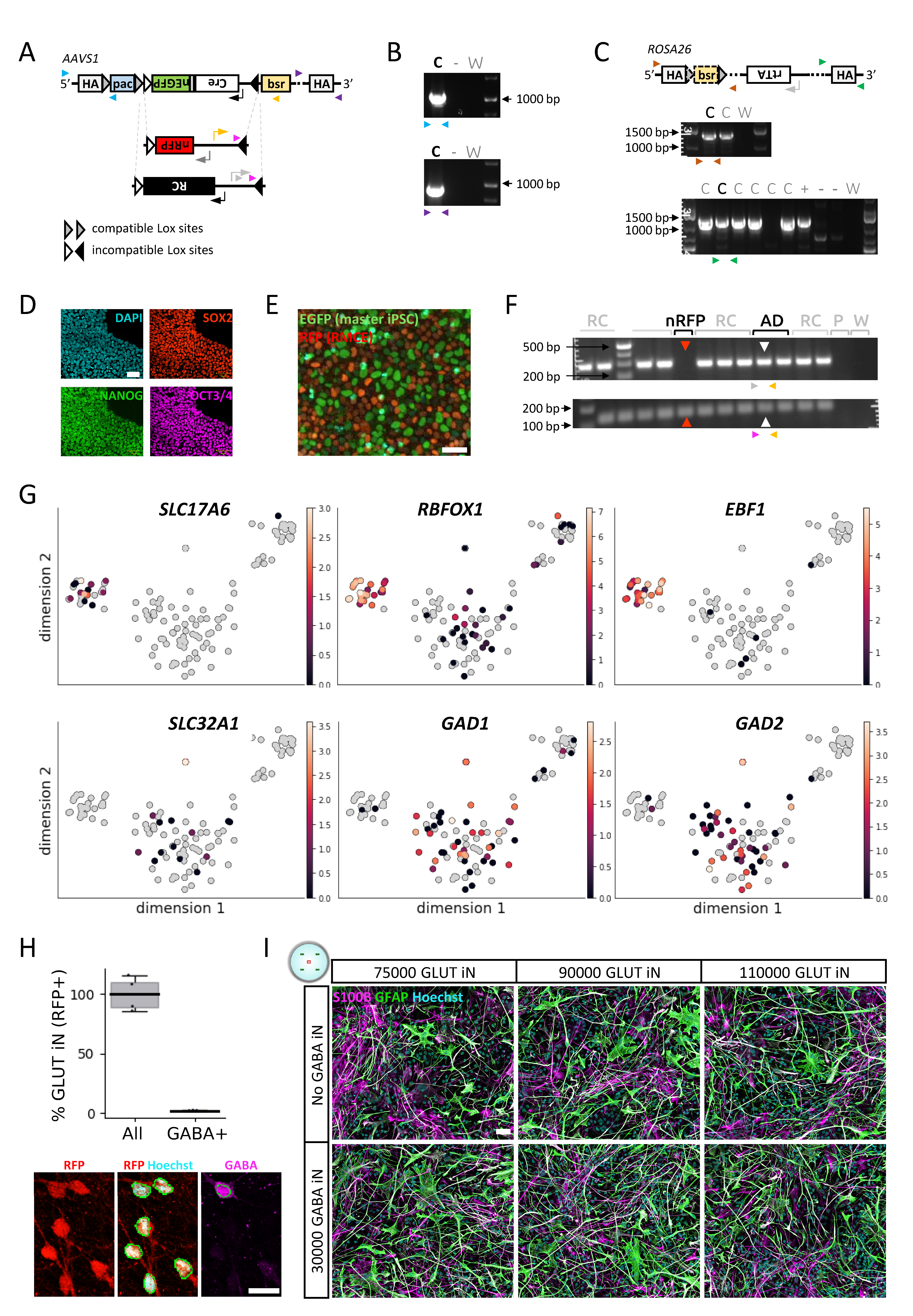

Supplement: Supplementary file 7 [file Image_4.TIF]

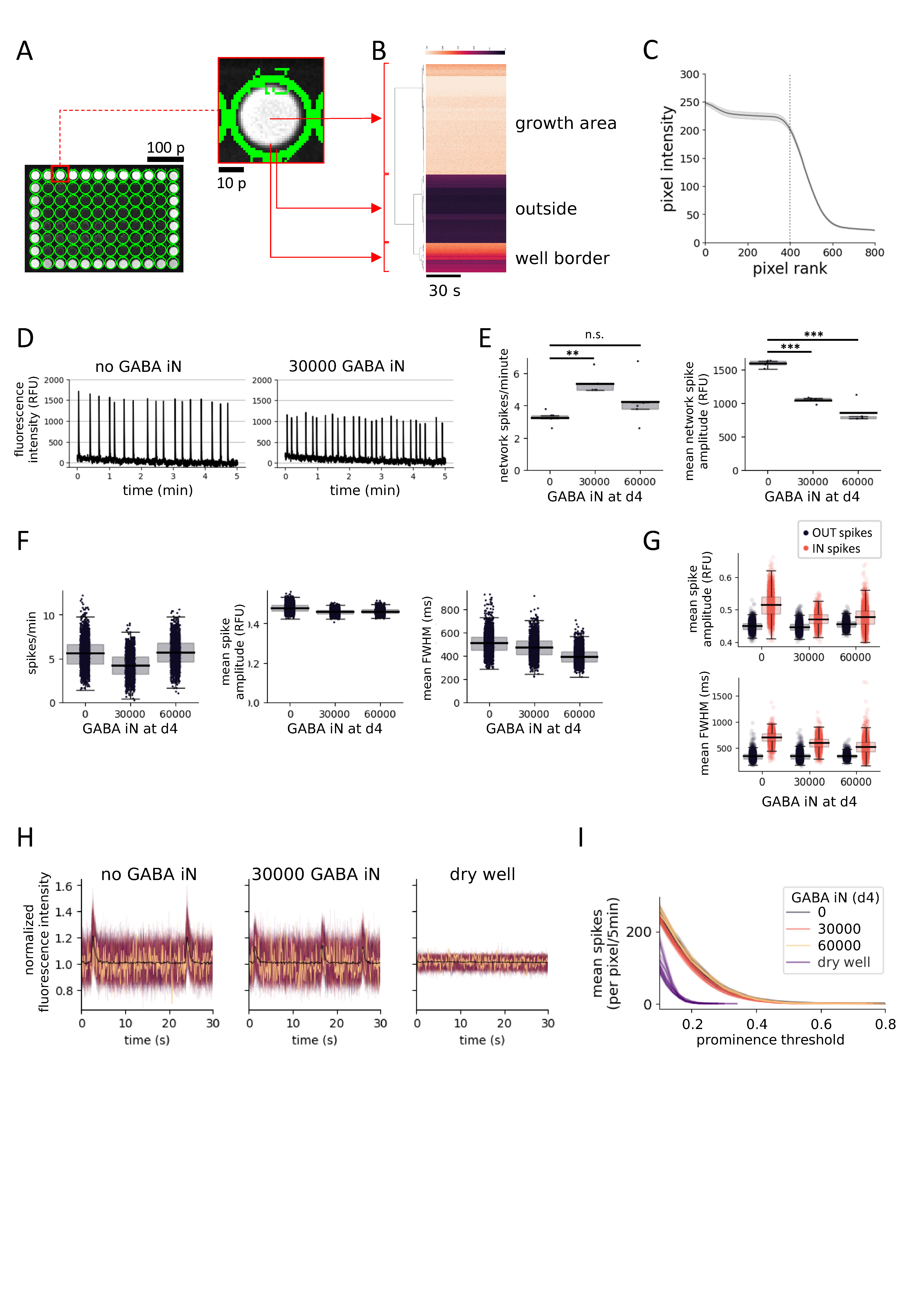

Supplement: Supplementary file 8 [file Image_5.TIF]

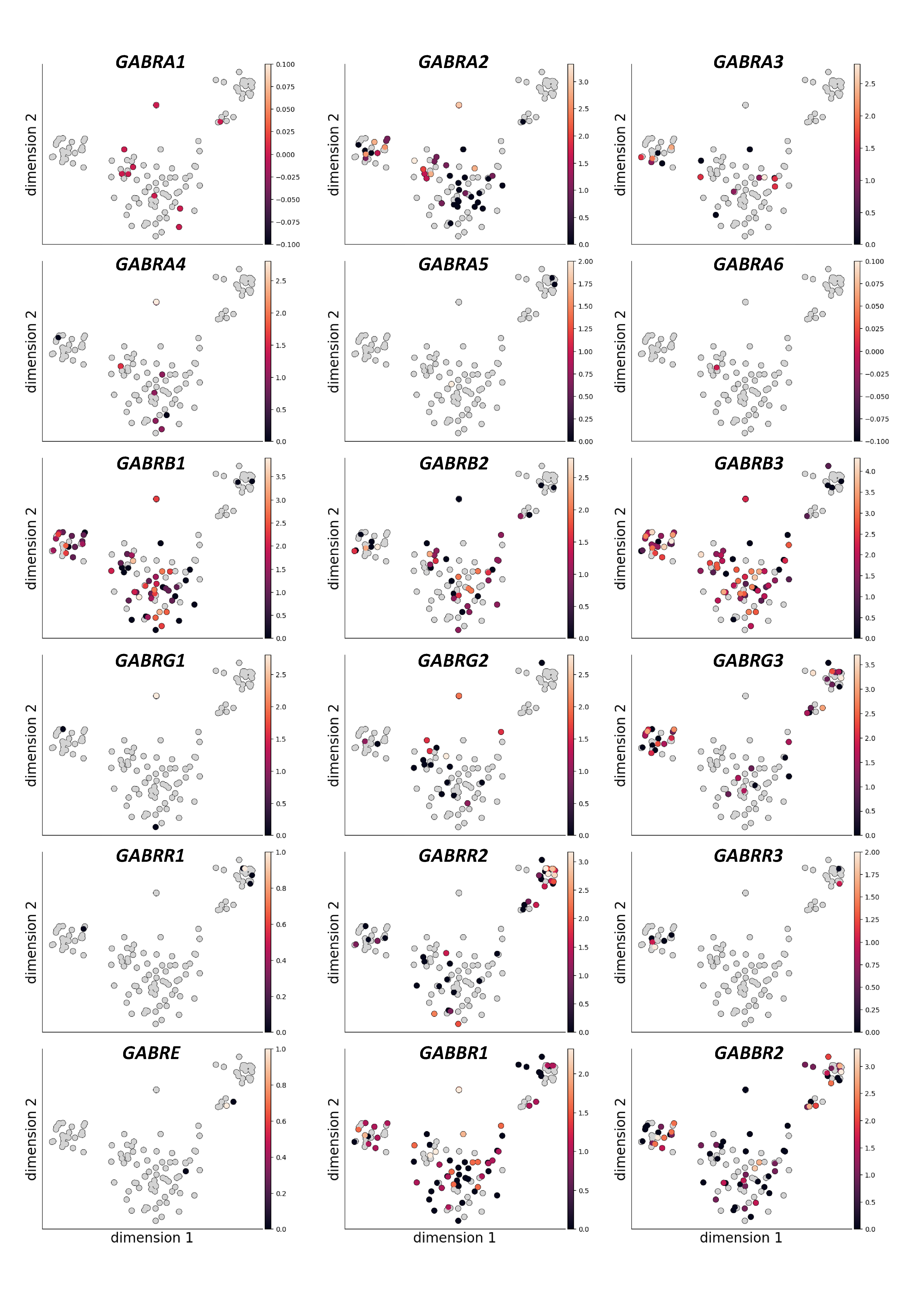

Supplement: Supplementary file 9 [file Image_6.TIF]

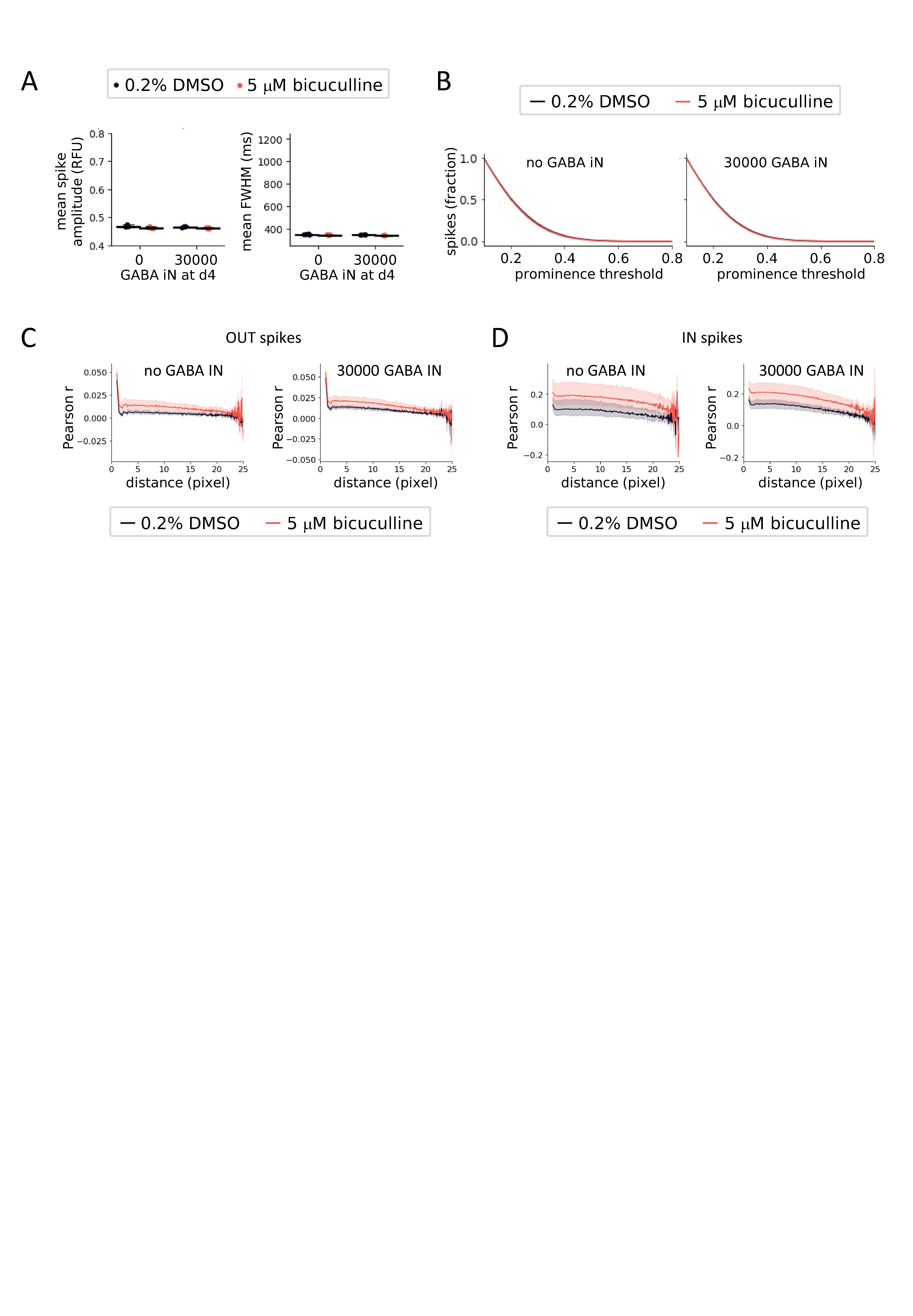

Supplement: Supplementary file 10 [file Image_7.TIF]
